# Club cell protein 16 (Cc16) deficiency increases inflamm‐aging in the lungs of mice

**DOI:** 10.14814/phy2.13797

**Published:** 2018-08-06

**Authors:** Maria E. Laucho‐Contreras, Francesca Polverino, Joselyn Rojas‐Quintero, Xiaoyun Wang, Caroline A. Owen

**Affiliations:** ^1^ Division of Pulmonary and Critical Care Medicine Brigham and Women's Hospital Harvard Medical School Boston Massachusetts; ^2^ The Lovelace Respiratory Research Institute Albuquerque New Mexico

**Keywords:** Club cell protein‐16, emphysema, inflammation, nuclear factor‐*κ*B, small airway fibrosis

## Abstract

Low serum CC16 levels are associated with accelerated lung function decline in human population studies, but it is not known whether low serum CC16 levels contribute to lung function decline, or are an epiphenomenon. We tested the hypothesis that unchallenged *Cc16* ^−/−^ *mice* develop accelerated rates of pulmonary function test abnormalities and pulmonary pathologies over time compared with unchallenged WT mice. Respiratory mechanics, airspace enlargement, and small airway fibrosis were measured in unchallenged wild‐type (WT) versus *Cc16* ^−/−^ mice over 6–18 months of age. Lung leukocyte counts and lung levels of metalloproteinases (Mmps), cytokines, oxidative stress, cellular senescence markers (p19 and p21), and lung cell apoptosis, and serum C‐reactive protein (CRP) levels were measured in age‐matched WT versus *Cc16* ^−/−^ mice. Unchallenged *Cc16* ^−/−^ mice developed greater increases in lung compliance, airspace enlargement, and small airway fibrosis than age‐matched WT mice over 6–18 months of age. *Cc16* ^−/−^ mice had greater: (1) lung leukocyte counts; (2) lung levels of Ccl2, Ccl‐5, interleukin‐10, Mmp‐2, and Mmp‐9; (3) pulmonary oxidative stress levels, (4) alveolar septal cell apoptosis and staining for p16 and p21; and (5) serum CRP levels. Unchallenged *Cc16* ^−/−^ mice had greater nuclear factor‐*κ*B (NF‐*κ*B) activation in their lungs than age‐matched WT mice, but similar lung levels of secretory phospholipase‐A2 activity. *Cc16* deficiency in mice leads spontaneously to an accelerated lung aging phenotype with exaggerated pulmonary inflammation and COPD‐like lung pathologies associated with increased activation of NF‐ *κ*B in the lung. CC16 augmentation strategies may reduce lung aging in *CC16*‐deficient individuals.

## Introduction

Physiological aging in humans, in the absence of cigarette smoke or other exposures, induces changes in the lungs that are similar to those that occur in patients with chronic obstructive pulmonary disease (COPD) including airspace enlargement, small airway disease, and a decline in forced expiratory volume in 1 sec (FEV_1)_ (Janssens et al. 1999; Vaz Fragoso and Gill 2010; Sorino et al. 2012). The pulmonary pathologies that contribute to age‐related decline in lung function over time in humans have not been well studied. In addition, some individuals have low FEV_1_ values by early adulthood, and others have accelerated lung function decline after 40 years of age (Lange et al. 2015), but the factors that contribute to these processes are not well understood. As‐yet‐unidentified circulating soluble factors reverse signs of aging in several tissues in elderly mice (Conboy et al. 2005), but circulating factors that regulate lung aging have not been identified in humans.

Club cell protein 16 (CC16; also known as uteroglobin, CC10, secretoglobin 1A1, Club cell secretory protein [CCSP]) is a circulating protein, and a member of the secretoglobin superfamily. CC16 is mainly expressed by Club cells in the airway epithelium, and is secreted by Club cells into the epithelial lining fluid and it readily diffuses into the circulation. CC16 has anti‐inflammatory and cytoprotective activities on various cell types including epithelial cells and leukocytes, and protects lungs from developing lung inflammation and injury mediated by allergens, viruses, bleomycin, and cigarette smoke (Chen et al. 2001; Wang et al. 2003; Lee et al. 2006; Snyder et al. 2010; Laucho‐Contreras et al. 2015a; Zhu et al. 2015). Low serum and lung levels of CC16 have been linked to the development of COPD, rapid decline in FEV_1_ in several human COPD studies (Bernard et al. 1992; Pilette et al. 2001; Lomas et al. 2008; Park et al. 2013; Laucho‐Contreras et al. 2015a; Petersen et al. 2015).

Recent human population studies have shown that, across the human adult lifespan, individuals with low serum CC16 levels have accelerated lung function decline compared with subjects with higher serum CC16 levels after correcting for covariates. Guerra et al. analyzed circulating CC16 levels measured at baseline in three population cohorts of adult subjects without COPD at baseline (>1600 adults aged 18–70 years in the United States and Europe) that had been followed longitudinally for 8–14 years (Celli and Owen 2013; Guerra et al. 2015). This study linked low baseline circulating CC16 levels to accelerated rates of FEV_1_ decline, the subsequent development of airflow obstruction (FEV_1_/forced vital capacity (FVC) < 70% predicted), and incident GOLD stage II COPD after adjusting for sex, age, height, smoking status, pack‐years smoking history, the presence of asthma, and FEV_1_ at baseline (Celli and Owen 2013; Guerra et al. 2015). However, it is not clear whether low circulating CC16 levels contribute directly to accelerate lung function decline in human, or whether they are an epiphenomenon. Also, the nature of the pulmonary pathologies underlying the lung function abnormalities were not evaluated in these human population studies.

*Cc16*‐deficient (*Cc16* ^−/−^) mice have normal lung development, and we reported previously that unchallenged young adult *Cc16* ^−/−^ mice (~12 weeks of age) have normal lung architecture (Laucho‐Contreras et al. 2015a). However, unchallenged 12‐week‐old *Cc16* ^−/−^ mice have higher lung leukocyte counts than age‐matched unchallenged WT mice indicating *Cc16* deficiency is sufficient to induce low‐grade pulmonary inflammation in young adult mice (Laucho‐Contreras et al. 2015a; Zhu et al. 2015). Thus, we hypothesized that *Cc16* deficiency (in the absence of any challenge) induces low‐grade chronic pulmonary inflammation which increases over time and contributes to accelerated lung aging characterized by COPD‐like lung pathologies to explain the accelerated lung function decline that is associated with low serum CC16 levels across the human lifespan (Guerra et al. 2015). To test this hypothesis, we compared the pulmonary phenotypes, and a readout of systemic inflammation (serum C‐reactive protein [CRP] levels), in WT and *Cc16* ^−/−^ mice that were housed in the absence of any challenge until they were 6, 12, or 18 months of age to model young adulthood, middle age, and late middle age, respectively, in humans (Demetrius 2006; Dutta and Sengupta 2016). Our results show that *Cc16* deficiency is sufficient to induce a low‐grade pulmonary inflammatory response in the lung which is associated with the development of airspace enlargement and small airway fibrosis, higher lung compliance, increased expression of markers of senescence in the lungs, and increased serum CRP levels in mice. In addition, we link exaggerated pulmonary lesions in unchallenged *Cc16* ^−/−^ mice to increased activation of NF‐*κ*B in the lung.

## Materials and Methods

### Animals

The Brigham and Women's Hospital Institutional Animal Care and Use Committee approved all procedures performed on mice. Animal experiments were conducted following ARRIVE guidelines. C57BL/6 *Cc16* ^−/−^ mice were generated as described previously (Stripp et al. 2002). Unchallenged *Cc16* ^−/−^ mice have a normal lifespan, fertility, and lung development (Stripp et al. 2002; Laucho‐Contreras et al. 2015a). Breeding pairs of C57BL/6 strain wild‐type (WT) mice were obtained from The Jackson Laboratory (Bar Harbor, ME), and housed in the same room in a barrier facility under specific pathogen‐free conditions as *Cc16* ^−/−^ breeding pairs. The WT and *Cc16* ^−/−^ progeny were used in all experiments. After weaning, male and female WT and *Cc16* ^−/−^ pups were housed in the same room and fed the same diet in the barrier facility for 6, 12, or 18 months in the absence of any challenge. The genotypes of the *Cc16* ^−/−^ mice were confirmed using PCR‐based protocols performed on genomic DNA extracted from tail biopsies.

### Respiratory mechanic in mice

Mice were anesthetized with 100 mg/kg ketamine, 10 mg/kg xylazine, and 3 mg/kg acepromazine, and a tracheostomy was performed. Respiratory mechanics were measured on the mice using a tracheal cannula connected to a digitally controlled mechanical ventilator (FlexiVent device; Scireq Inc., Montreal, QC, Canada) using the following settings: *f *=* *150/min; FiO_2_ = 0.21; tidal volume = 10 mL/kg body weight; and positive end‐expiratory pressure (PEEP) of 3 cm H_2_0, as described previously (Laucho‐Contreras et al. 2015b). Briefly, the lungs were inflated to total lung capacity (TLC; 25 cm H_2_O) three times, and then tissue and peripheral airway resistance (*G*), tissue and peripheral airway elastance (*H*), and peripheral airway dynamic compliance were measured using 3 cm H_2_O PEEP. The animals were then euthanized, and lungs were inflated to 25 cm H_2_O pressure and fixed for 18 h in 10% buffered formalin, or removed and frozen to −80°C.

### Airspace size

To quantify the distal airspace size in mice, alveolar chord lengths were measured on Gill's‐stained lung sections as described previously (Laucho‐Contreras et al. 2015b). Briefly, images of all well‐inflated microscopic fields of sections of both lung fields in each animal (~10 fields per animal) were captured (at ×100 magnification) using a Leica epi‐fluorescence microscope, a 3‐CCD color vision camera module, and Leica Qwin V3 software (Leica Microsystems Inc., Buffalo Grove, IL). Scion Image software (Scion Corp., Frederick, MD, USA) was used to measure alveolar chord length in microns (Laucho‐Contreras et al. 2015b).

### Small airway fibrosis

Small airway fibrosis was measured in inflated lung sections from unchallenged WT and *Cc16* ^−/−^ mice that were 6, 12, or 18 months of age. Formalin‐fixed paraffin‐embedded lung sections were stained with Masson's Trichrome stain using a commercial kit (Sigma‐Aldrich, St. Louis, MO). Images of both lung fields containing all airways having a diameter of 300–699 *μ*m in each mouse (10–20 airways/mouse) were acquired using microscope interfaced with a digital camera a camera. The thickness of the extracellular matrix protein layer (stained blue) around small airways having a mean diameter of 300–699 microns was measured using MetaMorph software (Molecular Devices, San Jose, CA), as described previously (Laucho‐Contreras et al. 2015b).

### Bronchoalveolar lavage leukocyte counts

Bronchoalveolar lavage (BAL) was performed on unchallenged WT and *Cc16* ^−/−^ and WT mice at 6, 12, or 18 months of age as described previously (Craig et al. 2013). BAL total leukocytes were counted, differential leukocyte counts were performed on modified Wright's‐stained cytocentrifuge preparations, and absolute numbers of leukocyte subsets were calculated.

### Lung levels of cytokines and growth factors

Levels of interleukin‐10 (Il‐10), Il‐6, Ccl5, Ccl2, and active transforming growth factor‐*β*_1_ (Tgf‐*β*_1_) were measured in homogenates of lung samples using ELISA kits (Peprotech, Rocky Hill, NJ; R&D Systems, Minneapolis, MN; and e‐Bioscience, San Diego, CA). All of the results were corrected for lung protein levels using a total protein assay kit (Thermo Fisher Scientific, Waltham, MA).

### Lung levels of matrix metalloproteinases

Levels of pro and active Mmp‐9 protein were measured in homogenates of lung samples obtained from unchallenged WT and *Cc16* ^−/−^ mice that were 6, 12, or 18 months of age using an ELISA kit (R&D Systems). The results were corrected for lung protein levels, using a total protein assay kit (Thermo Fisher Scientific, Waltham, MA).

Levels of active Mmp‐2 were measured in homogenates of lung samples using Western blotting after loading equal amounts of protein onto 12% SDS‐PAGE gels, separating the proteins by electrophoresis, and transferring the proteins to polyvinylidene difluoride membranes. Duplicate membranes were probed with either rabbit antimurine Mmp‐2 IgG diluted 1:1000 (Abcam, Cambridge, MA) or mouse antimurine alpha tubulin IgG (as a loading control) diluted 1:1000 (Abcam, Cambridge, MA) overnight at 4°C. After washing the membranes with PBS containing 0.1% Tween‐20, the membranes were incubated with horseradish peroxidase (HRP)‐conjugated goat antirabbit IgG or HRP‐conjugated goat antimurine IgG diluted 1:3000 (BioRad, Hercules, CA) for 1 h at room temperature. Signals were developed using a chemiluminescence kit (Thermo Scientific, Waltham, MA) following the manufacturer's instructions. Images of the signals were captured using a CCD camera, a ChemiDoc™ XRS+ System, and Image Lab software (Bio‐Rad Laboratories, Hercules, CA). The bands corresponding to active Mmp‐2 were analyzed using densitometry with ImageJ software (National Institutes of Health, Bethesda, MD) and signals for proMMP‐2 were normalized to alpha tubulin signals.

### Lung cell apoptosis

Cytoplasmic and nuclear fractions were extracted from frozen lung tissue obtained from unchallenged WT and *Cc16* ^−/−^ mice that were 6, 12, or 18 months of age using a commercial kit (Thermo Fisher Scientific, Waltham, MA). Cytoplasmic fractions and known standards of recombinant active caspase‐3 (BD Bioscience, San Jose, CA) were incubated in duplicate with a quenched fluorogenic substrate that is specific for active caspase‐3 (BD Bioscience, San Jose, CA) for 30 min at 37°C, protected from light. Cleavage of the substrate by active caspase‐3 in the samples was measured using fluorometry (with a F2500 fluorescence spectrophotometer; Hitachi, Tokyo, Japan; Ex *λ* 400 and Em *λ* 490). Concentrations of active caspase‐3 levels were calculated in fluorescence units by interpolation from the standard curve of active caspase‐3 exactly as described previously (Knolle et al. 2013).

### Staining of lungs for 4‐hydroxy‐2‐nonenal (4‐HNE)

Formalin‐fixed sections of lung obtained from unchallenged WT and *Cc16* ^−/−^ mice that were 6, 12, or 18 months of age (*n* = 3–4 mice/group) were incubated with blocking buffer followed by rabbit IgG to 4‐HNE (a marker of oxidative stress; Bioss, Woburn, MA) diluted 1:50 or nonimmune rabbit IgG at the same concentration for 1 h at 37°C. After washing the lung sections with PBS, the lung sections were incubated at 37°C for 1 h with Alexa 488‐conjugated goat antirabbit F(ab’)_2_ diluted 1:100. Sections were then washed in PBS, and nuclei were counterstained with 4′,6‐diamidino‐2‐phenylindole (DAPI). Images of the immunostained lung sections were captured using an epi‐fluorescence microscope and a digital camera. The number of 4‐HNE‐positive cells in the alveolar walls was counted and normalized to alveolar wall areas measured using MetaMorph software.

### Secretory phospholipase A_2_ (sPLA_2_) activity levels:

sPLA_2_ activity was measured in homogenates of whole lung samples obtained from unchallenged WT and *Cc16* ^−/−^ mice that were 6, 12, or 18 months of age using a commercially available kit (Enzo Life Science Inc., Farmingdale, NY). The results were normalized to total protein levels measured using a commercial total protein assay kit (Thermo Fisher Scientific, Waltham, MA.

### P16 and p21 staining in alveolar septal cells

Formalin‐fixed inflated lung sections obtained from unchallenged *Cc16* ^−/−^ and WT mice that were 6, 12, or 18 months of age were immunostained for two cyclin‐dependent kinases inhibitors (CDKI), p16 and p21, which are markers of cellular senescence (Wu et al. 2016). The sections were incubated with blocking buffer followed by rabbit anti‐p16 IgG, rat anti‐p21 IgG (Abcam, Cambridge, MA), nonimmune rabbit IgG (Dako North America Inc, Carpinteria, CA), or nonimmune rat IgG (Sigma‐Aldrich, St. Louis, MO). The sections were washed in PBS and then incubated with Alexa‐488‐conjugated goat antirabbit F(ab’)_2_ or goat antirat F(ab’)_2_ (Life Technology, Grand Island, NY). Images of the lung sections were captured using an epifluorescence microscope and a digital camera (Leica DFC300, Allendale, NJ). The numbers of alveolar septal cells that were positively stained for p16 or p21 were counted, and normalized to unit area of alveolar wall measured in pixels (Laucho‐Contreras et al. 2015aa) using MetaMorph software (Molecular Devices, Sunnyvale, CA).

### Nuclear Factor kappa B (NF‐*κ*B) activation in lung samples

NF‐*κ*B activation was measured in nuclear extracts prepared from whole lung samples obtained from unchallenged *Cc16* ^−/−^ and WT mice that were 6, 12, or 18 months of age using electrophoretic mobility shift assays (EMSA; Thermo Fisher Scientific, Waltham, MA) as described previously (Laucho‐Contreras et al. 2015a). Nuclear fractions were extracted from frozen lung tissue obtained from unchallenged mice using a commercial kit (Thermo Fisher Scientific, Waltham, MA). Protein levels in the cytoplasmic fractions were quantified using a commercial kit (Thermo Fisher Scientific, Waltham, MA). Equal amounts of protein (2 *μ*g) measured in the nuclear extracts were incubated with a biotin‐labeled probe having the consensus sequence to which NF*κ*B binds (sense strand: *5′ AGT TGA GGG GAC TTT CC CAG GC*) with or without excess unlabeled probe as a control, and subjected to gel electrophoresis for 3 h at 100 Volts. DNA:NF*κ*B complexes were transferred onto positive nylon membranes, probed with streptavidin–HRP, and signals were developed using a chemiluminescence kit (Thermo Fisher Scientific, Waltham, MA). Images of the blots were captured using a ChemiDoc™ XRS+ System, and Image Lab software (Bio‐Rad laboratories, Hercules, CA). Signals were quantified using densitometry with Image J software (National Institutes of Health, Bethesda, MD). It was not possible to run all of the samples (6 experimental conditions with 5 mice per experimental condition and controls) on one gel. Multiple gels were run on different days and samples from WT and *Cc16* ^−/−^ mice at the same time point were always analyzed on the same gels. As signal development time varied between gels, signals from *Cc16* ^−/−^ samples were normalized to those of WT samples obtained from mice at the same time point analyzed on the same gel.

### C‐reactive protein (CRP) levels in serum samples

Unchallenged WT and *Cc16* ^−/−^ mice that were 6, 12, or 18 months of age were euthanized, and blood samples were obtained via right ventricular puncture, and serum samples were frozen to −80°C. CRP levels were quantified in the serum samples using a commercially available ELISA kit (R&D Systems, Minneapolis, MN).

### Statistics

Statistical analyses were performed using SigmaPlot™ software (SSPS Inc, San Jose, USA). The Shapiro–Wilks test was performed to determine whether or not the data were normally distributed. Data were analyzed using one‐way analysis of variance and Holm–Sidak or Dunn post hoc methods for multiple group comparisons. Normally distributed data are presented as mean and SEM and pair‐wise testing was performed using Student's *t* tests. Data that were not normally distributed are presented as box‐plots showing medians and interquartile ranges (IQR), and error bars representing the 10th and 90th percentiles and pair‐wise testing was performed using Mann–Whitney *U* tests.

## Results

### *Cc16* ^−/−^ mice spontaneously develop greater airspace enlargement, lower lung resistance, and higher lung compliance than WT mice over 18 months of age

**Figure 1:**
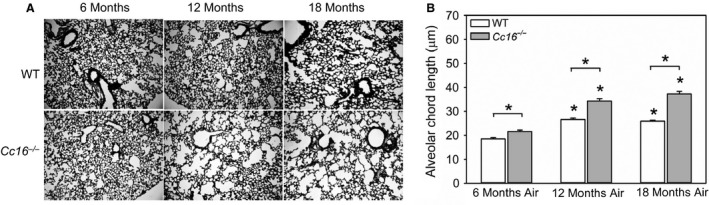
Greater airspace enlargement develops spontaneously in unchallenged *Cc16* ^−/−^ versus WT mice. (A) Gill's‐stained inflated lungs sections from unchallenged WT versus *Cc16* ^−/−^ mice at 6, 12, and 18 months of age that are representative of 8–12 mice per group. (B) Images of all well‐inflated microscopic fields of sections of both lung fields (~ten 100× microscopic fields per animal) were captured. Distal airspace size was measured as mean alveolar chord length in WT versus *Cc16* ^−/−^ mice at 6, 12, or 18 months of age using Scion image software, as described in Methods. The bars show the means and error bars show the SEM values. Data were analyzed using a one‐way ANOVA and pairwise testing was performed using Student’s *t*‐test. Asterisks indicate *P*  ≤  0.05 versus air‐exposed mice belonging to the same genotype at 6 months of age, or the group indicated; *n* = 9–14 mice/group.

None of the WT or *Cc16* ^−/−^ mice died during the 18 month of study period. Unchallenged WT and *Cc16* ^−/−^ mice that were 10 weeks of age had similar distal airspace size (Laucho‐Contreras et al. 2015a;). Unchallenged WT and *Cc16* ^−/−^ mice that were 12 or 18 months old had higher alveolar airspace size (measured as alveolar cord length) than 6‐month‐old mice belonging to the same genotype (Fig. 1). *Cc16* ^−/−^ mice had greater airspace enlargement than age‐matched WT mice at all time points tested. From 6 to 18 months of age, mean alveolar chord lengths increased to a greater extent in *Cc16* ^−/−^ mice (1.7‐fold) than WT mice (1.4‐fold). There were no differences in emphysema development in males versus females belonging to the same genotype at the same time point (data not shown).

Unchallenged 18‐month‐old WT mice had lower tissue and peripheral airway elastance values; lower tissue and peripheral airway resistance values; and higher peripheral airway compliance values than unchallenged 6‐month‐old WT mice (Table 1). Unchallenged 18‐month‐old *Cc16* ^−/−^ mice had lower tissue and peripheral airway elastance values; lower airway peripheral resistance values; and higher peripheral airway compliance values than unchallenged 6‐month‐old *Cc16* ^−/−^ mice (Table 1). Unchallenged *Cc16* ^−/−^ mice that were 6 months of age had lower tissue and peripheral airway elastance; lower peripheral airway resistance; and higher peripheral airway compliance than unchallenged WT mice that were 6 months old. Unchallenged *Cc16* ^−/−^ mice that were 18 months of age had lower tissue and peripheral airway resistance; and higher peripheral airway compliance than unchallenged WT mice that were 18 months old (Table 1). Thus, from 6 to 18 months of age, WT and *Cc16* ^−/−^ mice both developed airspace enlargement associated with increased lung compliance consistent with loss of elastic recoil in the lung. However, at 6 and 18 months of age, *Cc16* ^−/−^ mice developed greater airspace enlargement and had more compliant lungs than age‐matched WT mice.

**Table 1:** Respiratory mechanics in 6 and 18‐month‐old WT and *Cc16* ^−/−^ mice

Parameter measured	6 Months of age		18 Months of age	
	WT *n* = 11	*Cc16* ^−/−^ *n* = 11	WT *n* = 12	*Cc16* ^−/−^ *n* = 13
	Mean (SEM) or Median (IQR)	Mean (SEM) or Median (IQR)	Mean (SEM) or Median (IQR)	Mean (SEM) or Median (IQR)
Tissue elastance (cmH_2_O/mL)	19.3 (0.6)	17.1 (0.6)^a^	15.6 (0.7)^b^	15.1 (0.5)^c^
Peripheral elastance (cmH_2_O/mL)	21.0 (18.7–22.5)	18.1 (16.9–19.4)^a^	17.1 (15.4–19.0)^b^	16.3 (15.7–17.9)^c^
Tissue resistance (cmH_2_O/mL)	3.7 (0.3)	2.9 (0.2)^a^	3.0 (0.2)^b^	2.3 (0.07)^d^
Peripheral resistance (cmH_2_O/s/mL)	0.5 (0.02)	0.40 (0.01)	0.39 (0.02)^b^	0.31 (0.01)^c^^,^ d
Peripheral compliance (mL/cmH_2_O)	0.047 (0.002)	0.054 (0.002)^a^	0.057 (0.003)^b^	0.061 (0.002)^c^^,^ d

Respiratory mechanics were measured in unchallenged WT and *Cc16* ^−/−^ mice at 6 and 18 months of age in the using a FlexiVent device.

Data are mean ± SEM for data that are normally distributed or medians and interquartile ranges (IQR) for data that are not normally distributed (peripheral elastance values). Data were analyzed with ANOVAs (*P* < 0.001 for all parameters measured). Pair‐wise comparisons were performed using Student's *t*‐tests for data that are normally distributed or the Mann–Whitney *U* tests for data that are not normally distributed.

a *P* < 0.05 between 6‐month‐old WT and *Cc16* ^−/−^ mice.

b *P* < 0.05 between 6‐ and 18‐month‐old WT mice.

c *P* < 0.05 between 6‐ and 18‐month‐old *Cc16* ^−/−^ mice.

d *P* < 0.05 between 18‐month‐old WT and *Cc16* ^−/−^ mice.

### Unlike WT mice, *Cc16* ^−/−^ mice spontaneously develop small airway fibrosis over 18 months of age

**Figure 2:**
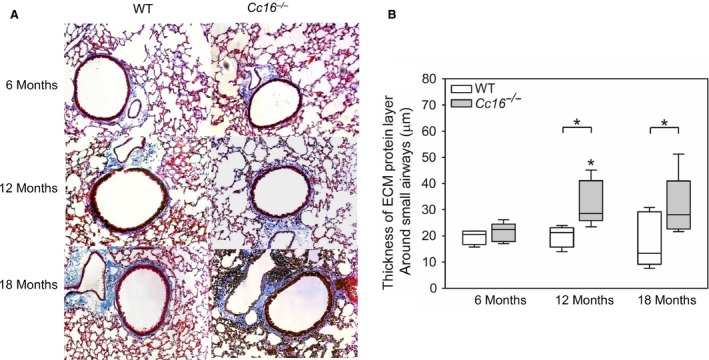
Small airway fibrosis develops over 18 months of age in unchallenged *Cc16* ^−/−^ mice but not in unchallenged WT mice. Deposition of extracellular matrix (ECM) proteins around small airways having a diameter of 300–699 microns was measured on Masson's Trichrome‐stained sections of lungs from unchallenged *Cc16* ^−/−^ versus WT mice at 6, 12, or 18 months of age. (A) Images of lung sections from mice that are representative of 5–9 mice/group at each time point. (B) Images of both lung fields containing all small airways having a diameter of 300–699 *μ*m in each mouse (10–20 airways per mouse) were acquired using a microscope interfaced with a digital camera a camera. The thickness of the ECM protein layer deposited around the small airways measured at 12 equally spaced points around each airway using MetaMorph software. The boxes in the box‐plots show the median values and 25th and 75th percentiles for the thickness of the ECM layer, and the whiskers show the 10th and 90th percentiles (5–9 mice/group). Data were analyzed using a one‐way ANOVA and pair‐wise testing was performed using Mann–Whitney *U* tests. Asterisk indicates *P* < 0.05 compared with mice belonging to the same genotype at 6 months of age, or the group indicated.

Unchallenged WT mice that were 12 or 18 months of age did not have greater small airway fibrosis (measured as deposition of extracellular matrix (ECM) around their small airways) compared with unchallenged WT mice that were 6 months of age (Fig. 2). In contrast, unchallenged 12‐ and 18‐month‐old *Cc16* ^−/−^ mice had greater small airway fibrosis than unchallenged 6‐month‐old *Cc16* ^−/−^ mice, but small airway fibrosis did not differ between 12‐ and 18‐month‐old *Cc16* ^−/−^ mice. *Cc16* ^−/−^ mice had greater small airway fibrosis both at 12 and 18 months of age (but not at 6 months of age) when compared with age‐matched WT mice (Fig. 2). From 6 to 18 months of age, *Cc16* ^−/−^ mice had a 1.3‐fold increase in the median value for small airway remodeling. There were no differences in small airway remodeling in males versus females belonging to the same genotype at the same time point (data not shown).

### *Cc16* ^−/−^ mice spontaneously develop greater lung inflammation than WT mice over 18 months of age

**Figure 3:**
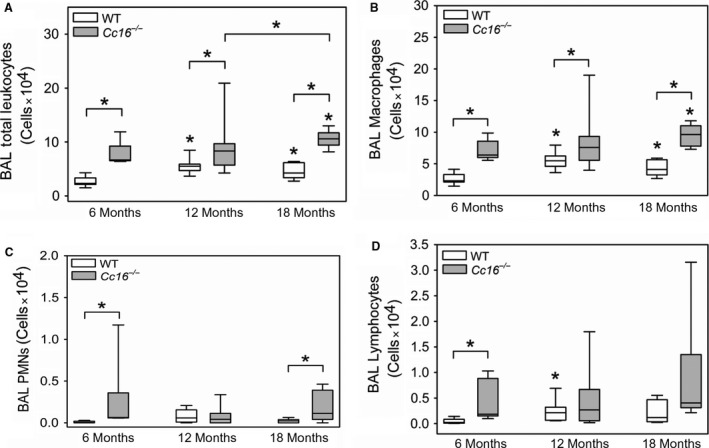
Greater pulmonary inflammation develops in unchallenged *Cc16* ^−/−^ versus WT mice. WT and *Cc16* ^−/−^ mice were housed for 6, 12, or 18 months in the absence of challenge. Bronchaolveolar lavage (BAL) was performed on each mouse, total leukocytes were counted with a hemocytometer, and differential leukocyte counts were performed on cytocentrifugre preparations that were stained with modified Wright's stain. Absolute numbers of leukocytes (A), macrophages (B), lymphocytes (C) and PMNs (D) were calculated. The boxes in the box‐plots show the median values and 25th and 75th percentiles, and the whiskers show the 10th and 90th percentiles (6–11 mice/group). Data were analyzed using one‐way ANOVAs and pairwise testing was performed using Mann–Whitney *U* tests. Asterisk indicates *P* < 0.05 compared with mice belonging to the same genotype at 6 months of age, or the group indicated.

Unchallenged WT mice and *Cc16* ^−/−^ mice at 12 and 18 months of age had higher BAL total leukocyte counts than mice belonging to the same genotype at 6 months of age, and BAL leukocyte counts were higher in 18‐month‐old versus 12‐month‐old *Cc16* ^−/−^ mice (Fig. 3A). BAL total leukocytes were higher in *Cc16* ^−/−^ mice than age‐matched WT mice at all time points tested. From 6 to 18 months of age, the median BAL total leukocyte counts increased 1.8‐fold in WT mice and 1.6‐fold in *Cc16* ^−/−^ mice. There were no differences in BAL total leukocyte counts at any time point in males versus females belonging to the same genotype at the same time point (data not shown).

Most of the leukocytes detected in BAL samples both WT and *Cc16* ^−/−^ mice were macrophages, and BAL macrophage counts were significantly higher in *Cc16* ^−/−^ mice than age‐matched WT mice at all time points tested (Fig. 3B). BAL PMN counts were higher in *Cc16* ^−/−^ mice at 6 and 18 months of age than age‐matched WT mice (Fig. 3C). BAL lymphocyte counts were higher in *Cc16* ^−/−^ mice 6 months of age than age‐matched WT mice, but not at later time points (Fig. 3D).

### *Cc16* ^−/−^ mice spontaneously develop higher lung levels of some cytokines than WT mice

**Figure 4:**
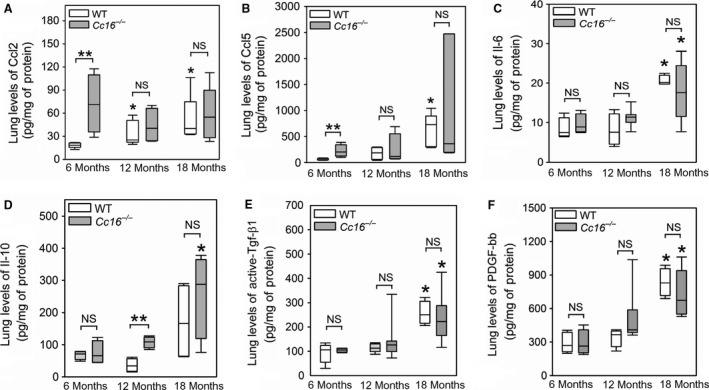
Higher levels of some proinflammatory chemokines and cytokines are detected in the lungs of unchallenged *Cc16* ^−/−^ versus WT mice. Levels of Ccl2 (A), and Ccl5 (B), interleukin‐6 (il‐6) (C), interleukin‐10 (D), transforming growth factor‐*β*1 (Tgf‐*β*1) (E); and platelet‐derived growth factor bb (PDGF‐bb) (F) were measured in homogenates of lung samples obtained from unchallenged WT and *Cc16* ^−/−^ mice at 6, 12, or 18 months of age using ELISAs, and values were normalized to lung total protein levels. In A–F, the boxes in the box‐plots show the median values and 25th and 75th percentiles, and the whiskers show the 10th and 90th percentiles (4–10 mice per group). Data were analyzed using one‐way ANOVAs and pair‐wise testing was performed using Mann–Whitney *U* tests. Asterisk indicates *P* < 0.05 compared with mice belonging to the same genotype at 6 months of age and **, *P* < 0.05 versus the groups indicated; NS, not significant.

Lung levels of two chemokines for monocytes and lymphocytes, Ccl2 and Ccl5, and proinflammatory (interleukin‐6) and anti‐inflammatory (interleukin‐10) cytokines were measured in WT and *Cc16* ^−/−^ mice. Lung levels of Ccl2 protein were higher in 12‐ and 18‐month‐old WT mice than 6 month of WT mice, but similar in *Cc16* ^−/−^ mice aged 6–18 months (Fig. 4A). Lung levels of Ccl2 protein were higher in 6‐month‐old *Cc16* ^−/−^ mice than 6‐month‐old WT mice, but similar in 12‐ and 18‐month‐old *Cc16* ^−/−^ mice and age‐matched WT mice. Lung levels of Ccl5 protein were higher in 18‐month‐old WT mice than 6‐month‐old WT mice (Fig. 4B), but similar in *Cc16* ^−/−^ mice that were 6–18 months of age. Lung levels of Ccl5 protein were higher in 6‐month‐old *Cc16* ^−/−^ mice than 6‐month‐old WT mice, but similar in 12‐ and 18‐month‐old *Cc16* ^−/−^ mice and age‐matched WT mice. Lung levels of interleukin‐6 protein were higher in 18‐month‐old WT and *Cc16* ^−/−^ mice than 6‐month‐old mice belonging to the same genotype (Fig. 4C). However, age‐matched WT and *Cc16* ^−/−^ mice did not differ in lung levels of interleukin‐6 at any time point assessed. Lung levels of interleukin‐10 protein were similar in 6, 12, and 18 month old WT mice, but higher in 18‐month‐old *Cc16* ^−/−^ mice than 6 month *Cc16* ^−/−^ mice (Fig. 4D). Lung levels of interleukin‐10 were higher in 12‐month‐old *Cc16* ^−/−^ mice than 12–month‐old WT mice, but were similar in WT and *Cc16* ^−/−^ mice that were 18 months old.

Lung levels of a profibrotic mediator that have been linked to small airway fibrosis (active Tgf‐*β*1 and Pdgf‐bb (Churg et al. 2006; Dai et al. 1998)) were also measured. Lung levels of active Tgf‐*β*1 and Pdgf‐bb protein were higher in 18‐month‐old WT mice than 6‐ or 12‐month‐old WT mice (Fig. 4E–F). Lung levels of active Tgf‐*β*1 protein were higher in 18‐month‐old *Cc16* ^−/−^ mice than 6‐ and 12‐month‐old *Cc16* ^−/−^ mice. However, lung levels of either these growth factors did not differ in *Cc16* ^−/−^ mice and age‐matched WT mice at any time point studied.

### *Cc16* ^−/−^ mice spontaneously develop higher lung levels of some matrix metallo‐proteinases than WT mice

**Figure 5:**
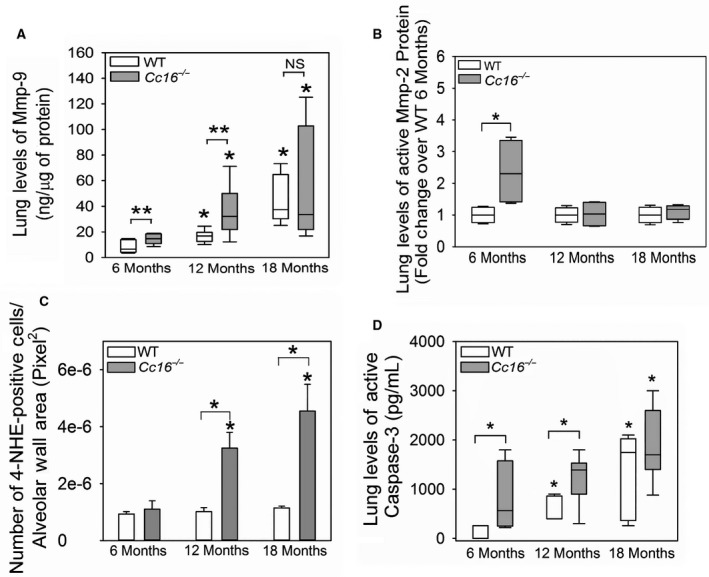
Greater lung levels of matrix metalloproteinases (Mmps) and oxidative stress and greater lung cell apoptosis develop in unchallenged *Cc16* ^−/−^ versus WT mice. Lung levels of pro and active Mmp‐9 (A) and active Mmp‐2 (B) were measured in homogenates of lung samples from unchallenged WT and *Cc16* ^−/−^ mice at 6, 12, or 18 months of age using ELISAs (5–9 mice/group) or western blotting (4 mice per group), respectively, and values were normalized to lung total protein levels. In (C), sections of inflated lungs harvested from 6‐, 12‐, or 18‐month‐old WT and *Cc16* ^−/−^ mice (3 mice/group) were immunostained with a green fluorophore for 4‐hydroxy‐nonenol (4‐HNE) as a marker of oxidative stress and the number of 4‐HNE‐positively stained cells was counted per unit area of alveolar walls (measured in pixels^2^). In (D), active caspase‐3 levels cytoplasmic fractions of whole lung samples from unchallenged WT and *Cc16* ^−/−^ mice at 6, 12, or 18 months of age (6–9 mice/group) using a quenched fluorogenic substrate that is specific for active capsase‐3, as described in Methods. In (A, B, and D), the boxes in the box‐plots show the median values and 25th and 75th percentiles, and the whiskers show the 10th and 90th percentiles. Data were analyzed using a one‐way ANOVA, and pairwise testing was performed using Mann–Whitney *U* tests. In (C), the bars represent the mean values and error bars represent SEM. Data were analyzed using a one‐way ANOVA, and pair‐wise testing was performed using Student's *t*‐tests. In (A–D), asterisk indicates *P* < 0.05 compared with mice belonging to the same genotype at 6 months of age, or the groups indicated; NS, not significant.

Metalloproteinases (Mmps) are produced by leukocytes and lung parenchymal cells and contribute to emphysema development and small airway fibrosis in experimental animals exposed to cigarette smoke (Hautamaki et al. 1997; Churg et al. 2007). Lung levels of Mmp‐2 and ‐9 also increase as mice age (Sueblinvong et al. 2012). Lung levels of Mmp‐9 protein (pro and active forms) were higher in unchallenged 12‐ and 18‐month‐old WT and *Cc16* ^−/−^ mice than unchallenged mice belonging to the same genotype at 6 months of age (Fig. 5A). Unchallenged *Cc16* ^−/−^ mice that were 6‐ and 12‐months of age had higher lung levels of pro‐Mmp‐9 than age‐matched unchallenged WT mice. Lung levels of active Mmp‐2 protein were higher in 6‐month‐old *Cc16* ^−/−^ mice than 6‐month‐old WT mice, but similar in 12‐month and 18‐month‐old *Cc16* ^−/−^ mice and age‐matched WT mice (Fig. 5B).

### *Cc16* ^−/−^ mice spontaneously develop greater lung oxidative stress levels than WT mice over 12–18 months of age

Pulmonary inflammation is associated with increases in lung oxidative stress levels (Owen 2005), and oxidative stress levels also increase in the aging lung and contribute to pulmonary inflammation and injury (Cannizzo et al. 2011; Sohal and Orr 2012). Lung oxidative stress levels [measured as staining for 4‐hydroxy‐2‐nonenal (4‐HNE) in the alveolar walls)] were similar in WT mice at 6, 12, and 18 months of age (Fig. 5C). However, 4‐NHE staining in the alveolar walls was higher in *Cc16* ^−/−^ mice that were 12 and 18 months of age than in *Cc16* ^−/−^ mice that were 6 months of age. *Cc16* ^−/−^ mice that were 12 and 18 months old had greater staining for 4‐HNE in their alveolar walls than aged‐matched WT mice. From 6 to 18 months of age the mean 4‐HNE staining in the alveolar walls increased to a greater extent in *Cc16* ^−/−^ mice (4.1‐fold) than WT mice (1.2‐fold).

### *Cc16* ^*−/−*^ mice spontaneously develop greater rates of lung cell apoptosis than WT mice over 18 months of age

Apoptosis of alveolar septal cells contributes to emphysema development in experimental murine models of COPD (Kasahara et al. 2000) and senile emphysema (Calvi et al. 2011). Lung levels of active caspase‐3 (a readout of lung cell apoptosis) were higher in 12‐ and 18‐month‐old WT mice than 6‐month‐old WT mice, and higher in 18‐month‐old *Cc16* ^−/−^ mice than 6‐month‐old *Cc16* ^−/−^ mice (Fig. 5D). Lung levels of active caspase‐3 were higher in *Cc16* ^−/−^ mice that age‐matched WT mice at 6 and 12 (but not at 18) months of age.

### *Cc16* ^−/−^ mice have greater staining for cyclin‐dependent kinases (CDK) inhibitors in alveolar septal cells than WT mice over 18 months of age

**Figure 6:**
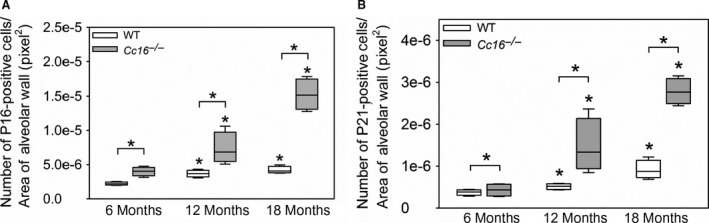
Greater expression of senescence markers (p16 and p21) in alveolar septal cells develops in unchallenged *Cc16* ^−/−^ versus WT mice. WT and *Cc16* ^−/−^ mice were housed in the absence of challenge for 6, 12, or 18 months. Fixed, inflated lungs sections from the animals were immunostained with a green fluorophore for p16 (A) or p21 (B), and the number of positively stained cells per unit of alveolar wall area (measured in pixels^2^) was quantified. The boxes in the box‐plots show the median values and 25th and 75th percentiles, and whiskers show the 10th and 90th percentiles; *n* = 4 mice/group. Data were analyzed using one‐way ANOVAs and pairwise testing was performed using Mann–Whitney *U* tests. Asterisk indicates *P* < 0.05 compared with mice belonging to the same genotype at 6 months of age, or the group indicated.

CDK inhibitors (including p16 and p21) are the main inducers of cell senescence (Karrasch et al. 2008). P21 initiates cellular senescence, and p16 maintains this process (Stein et al. 1999). Staining for both p16 and p21 in the alveolar walls was higher in 12‐ and 18‐month‐old WT and *Cc16* ^−/−^ mice than mice belonging to the same genotype at 6 months of age (Fig. 6). *Cc16* ^−/−^ mice had greater staining for both p16 and p21 in their alveolar walls than age‐matched WT mice at all time points assessed. From 6 to 18 months of age, median P16 staining in the alveolar walls increased to a greater extent in *Cc16* ^−/−^ mice (3.8‐fold) than WT mice (1.8‐fold). From 6 to 18 months of age, median p21 staining in the alveolar walls also increased to a greater extent in *Cc16* ^−/−^ mice (6.4‐fold) than WT mice (2.3‐fold).

### *Cc16* ^−/−^ mice have greater serum C‐reactive protein (CRP) levels than WT mice at 18 months of age

**Figure 7:**
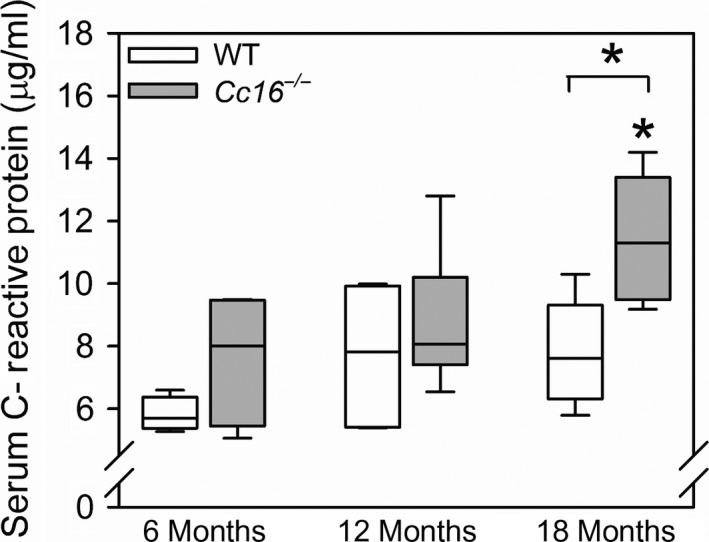
Serum C‐reactive protein (CRP) levels are higher in unchallenged *Cc16* ^−/−^ than WT mice at 18 months of age: Serum samples were obtained from unchallenged WT and *Cc16* ^−/−^ mice at 6 months of age (4 mice per group), 12 months of age (5–6 mice/group), or 18 months of age (5 mice/group) via cardiac puncture. Serum CRP levels were measured using an ELISA. The boxes in the box‐plots show the median values and 25th and 75th percentiles, and the whiskers show the 10th and 90th percentiles. Data were analyzed using a one‐way ANOVA followed by pairwise testing with Mann–Whitney *U* tests. Asterisk indicates 0.05 versus the group indicated or *Cc16* ^−/−^ mice at 6 months of age.

Systemic inflammation increases during the aging process (Guarner and Rubio‐Ruiz 2015) and serum CRP levels are a measure of systemic inflammation (Roubenoff et al. 1998). Serum CRP levels were higher in unchallenged 18‐month‐old *Cc16* ^−/−^ mice than unchallenged 6‐month‐old *Cc16* ^−/−^ mice, but were similar in unchallenged WT mice at 6, 12, and 18 months of age (Fig. 7). Serum CRP levels were higher in 18‐month‐old unchallenged *Cc16* ^−/−^ mice than 18‐month‐old unchallenged WT mice, but serum CRP levels did not differ between WT and *Cc16* ^−/−^ mice that were 6 or 12 months of age (Fig. 7). From 6 to 18 months of age, there was a 1.4‐fold increase in the median serum CRP levels in *Cc16* ^−/−^ mice.

### *Cc16* ^−/−^ mice have greater NF‐kB activation in their lungs than WT mice, but similar lung levels of sPLA_2_

**Figure 8:**
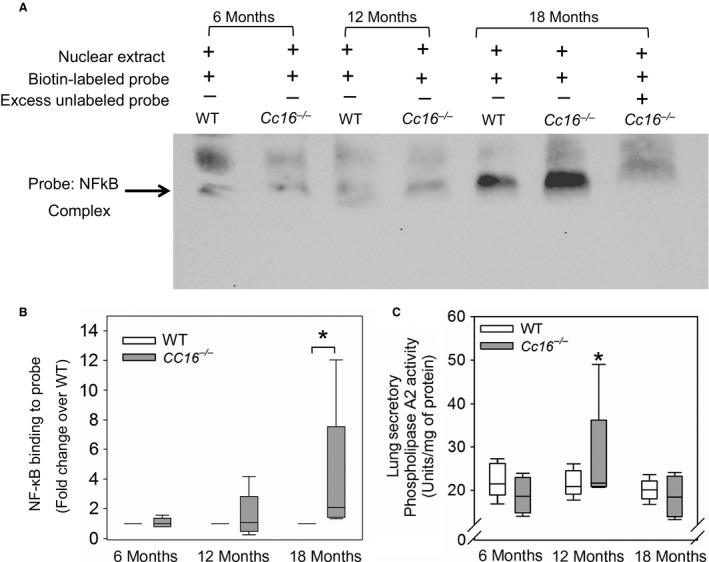
Greater NF‐kB activation occurs in whole lung samples from unchallenged *Cc16* ^−/−^ mice versus WT mice at 18 months of age. NF *κ*B activation was measured in nuclear extracts prepared from whole lung samples from unchallenged *Cc16* ^−/−^ and WT mice at 6, 12, or 18 months of age using an electrophoretic mobility shift assay (EMSA) with a biotin‐labeled oligonucleotide probe having the consensus sequence to which the NF *κ*B transcription factor binds, as described in Methods. (A) An EMSA gel is shown which is representative of five animals for each experimental condition. The last lane on the right of the gel shows a control in which an excess of the unlabeled oligonucleotide probe was added along with the biotin‐labeled probe to the nuclear extract from an aliquot of the sample prepared from the 18‐month‐old *Cc16* ^−/−^ mouse, as a control. Loss of the signal corresponding to the NF *κ*B‐oligonucleotide complex in the last lane indicates that the NF *κ*B present in the nuclear extract binds specifically to the biotin‐labeled probe. (B) The intensities of the bands corresponding to *NF‐* *k*B transcription factor bound to the oligonucleotide probe were quantified using densitometry (*n* = 5 mice per genotype per time point). As multiple gels had to be run to accommodate all of the samples, the band intensities for samples from *Cc16* ^−/−^ mice normalized to the mean value of the WT sample at the same time point analyzed on the same gel, as described in Methods. The boxes in the box‐plots show the median values and 25th and 75th percentiles, and whiskers show the 10th and 90th percentiles. Data were analyzed using a one‐way ANOVA and pair‐wise testing was performed using Mann–Whitney *U* tests. Asterisk indicates *P* < 0.05 versus the group indicated. (C) Lung levels of secretory phospholipase A_2_ activity were measured in unchallenged *Cc16* ^−/−^ and WT mice at 6, 12, or 18 months of age (*n* = 4–5 mice per group) using a kit. The boxes in the box‐plots show the median values and 25th and 75th percentiles, and the whiskers show the 10th and 90th percentiles. Data were analyzed using a one‐way ANOVA and pairwise testing was performed using Mann–Whitney *U* tests. Asterisk indicates *P* < 0.05 versus the *Cc16* ^−/−^ mice that were 6 months of age.

Cc16 has been shown to mediate its anti‐inflammatory activities by inhibiting activation of *NF‐kB* or inhibiting the catalytic activity of secretory phospho‐lipase A2 (*sPLA*_*2*_ *,* a proinflammatory pathway) (Laucho‐Contreras et al. 2016) in different model systems. *NF‐kB* activation (assessed using EMSA assays) was higher in nuclear extracts of whole lung samples from 18‐month‐old *Cc16* ^−/−^ mice than 18‐month‐old WT mice (Fig. 8A–B), but 6‐ and 12‐month‐old WT mice did not differ in levels of *NF‐kB* activation in their lungs. Whole lung levels of active sPLA_2_ were similar in *Cc16* ^−/−^ mice and aged‐matched WT mice at all time points assessed (Fig. 8C).

## Discussion

We report for the first time that unchallenged *Cc16* ^−/−^ mice develop greater increases in lung compliance and greater airspace enlargement than WT mice before the onset of senescence. Unlike WT mice, unchallenged *Cc16* ^−/−^ mice also developed small airway fibrosis by 12 months of age. The greater pulmonary inflammation and oxidative stress levels, and greater alveolar septal cell apoptosis and senescence that spontaneously developed in *Cc16* ^−/−^ versus age‐matched WT mice likely contributed to their exaggerated pulmonary pathologies. In addition, the greater activation of *NF‐kB* in the lungs of *Cc16* ^−/−^ mice may have contributed to the exaggerated pulmonary inflammation and alveolar septal cell perturbations detected in *Cc16* ^−/−^ mice, as NF‐kB is a transcription factor that promotes inflammation and senescence and has been strongly linked to aging in many tissues in rodents and humans (Adler et al. 2007). Our data suggest that the low circulating levels of CC16 levels that occur in a subset of human subjects (Celli and Owen 2013; Guerra et al. 2015) may contribute directly to the accelerated lung function decline detected in these subjects by accelerating lung inflamm‐aging and inducing airspace enlargement and small airway fibrosis.

Physiological aging leads to changes in the lung similar to those occurring in COPD patients, including airspace enlargement (“senile emphysema”), a decline in the FEV_1_(Verbeken et al. 1992; Janssens et al. 1999; Vaz Fragoso and Gill 2010), and small airway disease, as assessed using computed tomography scans of the thorax, spirometry, and multiple breath washout methods (Janssens et al. 1999; Verbanck et al. 2012; Bommart et al. 2015). However, the mechanisms involved in these normal aging‐related pulmonary pathologies have not been well studied. In addition, the pulmonary pathologies and mechanisms underlying the accelerated decline in lung function that occurs in some individuals during middle age (Lange et al. 2015) have been even less well studied.

Mice are commonly used to model the human aging processes in organs of interest as they are less expensive to house than larger animals and have senescence pathways which are similar to those in humans (Demetrius 2006; Dutta and Sengupta 2016). Six months of age in mice is equivalent to young adulthood (~20 years of age) in humans; 12 months of age in mice is equivalent to middle age (~40 years of age in humans); 18 months is defined as the beginning of senescence in mice (~60 years of age in humans) (Dutta and Sengupta 2016). We studied time points at or before the normal onset of senescence in mice to test our hypothesis that *Cc16* deficiency accelerates the lung aging process in mice, and leads to detectable pulmonary disease well before the onset of senescence. Furthermore, the period before middle age represents the optimal window in which novel therapies could be initiated to effectively prevent accelerated aging and preserve lung function.

Normal aging is associated with a low‐grade chronic inflammatory process or “inflamm‐aging” in the lungs and also in other organs including the kidney, cardiovascular and endocrine systems, and in skeletal muscle (Fougere et al. 2017; O'Sullivan et al. 2017). Herein, greater pulmonary inflammation along with higher serum levels of CRP were detected in *Cc16* ^−/−^ mice versus age‐matched WT mice suggesting that *Cc16* deficiency increases pulmonary and systemic inflammation. Future studies will assess whether deficiency of *Cc16* leads to accelerated aging in organs other than the lung.

Human cohort studies have linked low serum levels of CC16 to increased rate of FEV_1_ decline, reduced FEV_1_/FVC ratios, increased airway hyper‐responsiveness, and the development of moderate airflow obstruction in adults after the adjusting for sex, age, height, baseline FEV_1_, smoking status, and pack/years smoking history (Guerra et al. 2015; Rava et al. 2015). However, the lung pathologies underlying the increased rate of FEV_1_ and airflow obstruction that are associated with low serum CC16 levels in humans have not been assessed. Our study adds to this literature by showing that *Cc16* deficiency is sufficient to accelerate the spontaneous development of pulmonary inflammation and emphysema, and to induce small airway fibrosis over 18 months of age in mice. Thus, the increased rate of lung function decline that has been reported in humans with low serum CC16 levels (Guerra et al. 2015; Rava et al. 2015) may be due, in part, reduced CC16‐mediated protection from inflamm‐aging in the lung. Whether humans with low CC16 levels also have accelerated development of airspace enlargement and small airway fibrosis to explain their accelerated decline in FEV_1_ should be addressed by future lung imaging and/or studies of lung samples from humans with and without low serum CC16 levels.

### Emphysema development

In cigarette smoke (CS)‐exposed mice, pulmonary inflammation, increases in lung proteinase and oxidative stress levels, and alveolar septal cell apoptosis promote loss of the alveolar walls and emphysema development (Kasahara et al. 2000; Rangasamy et al. 2004; Owen 2005, 2008). There have been fewer studies of senile emphysema in mice. However, the lung phenotypes of DBA/2 mice (an inbred strain which exhibits accelerated aging) have been examined over 20 months of age (Calvi et al. 2011). Between 8 and 12 months of age, DBA/2 mice developed greater lung oxidative stress levels, higher rates of alveolar septal cell apoptosis, and higher lung levels of Mmp‐9 (but not Mmp‐12), followed by increases in lung leukocyte counts and greater emphysema development than DBA/2 mice that were 2–4 months of age. Another study reported that C57BL/6 strain WT mice that were 9 months old did not have greater airspace enlargement or higher tissue PMN or macrophage counts, lung oxidative stress levels, or *p21* or *p16* gene expression levels than 18‐month‐old C57BL/6 WT mice (Zhou et al. 2013). However, the latter study did not evaluate C57BL/6 mice that were younger than 9 months of age as controls. Our study showed that increased BAL leukocyte counts and airspace enlargement were detected from 6 to 12 months of age in C57BL/6 WT mice but these changes did not progress further by 18 months of age.

The increased lung macrophage counts, and greater alveolar septal cell apoptosis, lung oxidative stress levels, and staining for two senescence markers (p21 and p16) detected in unchallenged *Cc16* ^−/−^ mice likely contributed synergistically to the accelerated emphysema observed in these animals. Macrophages and their products (especially Mmp‐12) are required for the development of CS‐induced emphysema (Hautamaki et al. 1997; Ofulue and Ko 1999). Increases in lung macrophage counts (Takeda et al. 2008), Mmp expression by lung macrophages (Morris et al. 2003; Takeda et al. 2008; Xu et al. 2017), lung oxidative stress levels (Lao et al. 2016), alveolar septal cell apoptosis (Suga et al. 2000; Ishii et al. 2008), and the expression of senescence markers in the lung (Karrasch et al. 2008) have all been linked to the emphysema that develops spontaneously in gene‐targeted mice that develop an accelerated lung aging phenotype. It is possible that the increased lung macrophage counts detected in *Cc16* ^−/−^ lungs contributed (in part) to the higher lung burden of some of the mediators detected in unchallenged *Cc16* ^−/−^ mice (such as pro‐Mmp‐9 and ‐2, Ccl2, Ccl5, and interleukin‐10), as these mediators are produced by macrophages. The higher BAL lymphocyte counts detected in unchallenged *Cc16* ^−/−^ versus WT mice at 6 months of age may be due to the higher lungs levels of Ccl‐2 and Ccl‐5 detected at this age.

The increased lung levels of Mmp‐9 and Mmp‐2 detected in *Cc16* ^−/−^ mice likely contributed to increased lung ECM degradation and their increased loss of alveolar walls. The elevated lung levels of Ccl2 and Ccl5 may also have amplified the increased lung macrophage counts detected in *Cc16* ^−/−^ mice aged 6–18 months as both of these mediators are chemokines for monocytes (Owen et al. 1994). Surprisingly, lung levels of interleukin‐10 (an anti‐inflammatory cytokine) were higher *in Cc16* ^−/−^ mice than age‐matched WT mice, but were still insufficient to attenuate the increased lung inflammation detected in *Cc16* ^−/−^ mice at 6–18 months of age.

### Small airway fibrosis

Small airway fibrosis has been detected in older human subjects (Janssens et al. 1999; Verbanck et al. 2012; Bommart et al. 2015), and contributes significantly to airflow obstruction in COPD patients (Hogg et al. 2004). To our knowledge, there has been only one study of small airway fibrosis in unchallenged C57BL/6 WT mice older than 6 months of age. Zhou et al. reported that C57BL/6 WT mice aged 18 months did not have greater small airway fibrosis than C57BL/6 WT mice that were 9 months of age (Zhou et al. 2013). Our studies of this lung phenotype in WT mice agree with those of Zhou et al. *Cc16* deficiency spontaneously led to the development of small airway fibrosis that was detectable by 12 months of age. Small airway fibrosis in CS‐exposed rodents has been linked to increased generation of profibrotic growth factors including Tgf‐*β* (Churg et al. 2006), and proinflammatory mediators (Churg et al. 2007, 2009, 2012). Lung levels of active Tgf‐*β* were similar in *Cc16* ^−/−^ and WT mice at all time points assessed, but it is possible that Tgf‐*β* levels were higher in local microenvironments in *Cc16* ^−/−^ small airways. Whether the greater lung inflammation detected in *Cc16* ^−/−^ mice contributed to their exaggerated small airway fibrosis will be examined in future studies. Exogenous CC16 reduces the activation of fibroblast‐like cells in vitro (Antico et al. 2014). Whether the loss of Cc16‐mediated inhibition of fibroblast activation contributed to the accelerated development of small airway fibrosis in unchallenged *Cc16* ^−/−^ mice will be examined in our future studies.

Cc16 has potent anti‐inflammatory activities in numerous models of acute and chronic lung disease (Laucho‐Contreras et al. 2015a, 2016). The anti‐inflammatory activities of Cc16 in other models have been linked to its capacity to inhibit the catalytic activity of secretory phospholipase A_2_ (sPLA_2_), a potent proinflammatory pathway by binding to co‐factors required for full catalytic activity of this enzyme (Levin et al. 1986; Lesur et al. 1995), or inhibition of activation of NF*κ*B (Laucho‐Contreras et al. 2015a, 2016) depending on the model system studied. Herein, WT and *Cc16* ^−/−^ mice did not differ in lung sPLA_2_ activity levels. The greater NF‐*κ*B activation that was detected in the lungs of 18‐month‐old *Cc16* ^−/−^ versus age‐matched WT mice likely contributed to the greater lung inflammation and pulmonary lesions detected in unchallenged *Cc16* ^−/−^ *mice*. Greater (NF*κ*B) activation was not detected in *Cc16* ^−/−^ lungs at earlier time points despite the greater lung inflammation detected at 6–12 months of age in these mice. However, we measured NF‐*κ*B activation only in whole lung samples, and signals due to increased NF‐*κ*B activation in specific sub‐types of cells in the lung (e.g., inflammatory cells) at earlier time points may have been diluted out in EMSAs conducted on whole lung samples. How CC16 inhibits activation of NF‐kB is not known, and is currently an area of intense research investigations by a number of laboratories (Laucho‐Contreras et al. 2016).

Aging is thought to be induced by cumulative stochastic injury to proteins, DNA, mitochondria, and telomeres and other cellular components (Liu et al. 2002; Kirkwood 2005; Green et al. 2011) which is driven by oxidative stress (Packer and Fuehr 1977) generated by chronic inflammation (Franceschi et al. 2007; Green et al. 2011). Although cellular injury may cause aging by inducing loss of functional cells in tissues, the cellular response to injury may contribute significantly to the aging process. NF‐*κ*B activation is a key component of the cellular response to injury (Hayden and Ghosh 2008). NF‐*κ*B activation may drive cellular senescence in many tissues during normal and accelerating aging in mice and humans (Osorio et al. 2016). NF‐*κ*B is the transcription factor that is most highly associated with mammalian aging (Adler et al. 2007), and NF‐*κ*B activation is increased in most organs of old versus young rodents (Helenius et al. 1996; Korhonen et al. 1997). Chronic activation of NF‐*κ*B occurs in numerous human age‐related diseases including sarcopenia, atherosclerosis, osteoarthritis, dementia, osteoporosis, and COPD (Yamamoto and Gaynor 2001; Tilstra et al. 2011; Mercado et al. 2015).

Soluble factors present in the circulation regulate aging in mammals. In a murine model of heterochronic parabiosis (in which old and young mice are joined together surgically to share a circulatory system), Ccl11 was present at elevated levels in the old mice, and was shown to induce accelerated aging of the nervous system in the young mice (Villeda et al. 2011). As‐yet‐unidentified circulating soluble factors in old mice that bind and activate Frizzled receptor converted muscle stem cells from a myogenic to a fibrogenic lineage with reduced regenerative potential by activating Wnt signaling (Villeda et al. 2011). Another study of murine heterochronic parabionts reported that as‐yet‐unidentified circulating factors in young mice reversed the aging process in the skeletal muscle and livers of the old mice (Conboy et al. 2005), but the lungs were not examined in this study. Whether CC16 is one such circulating protein that restrains inflamm‐aging in the lung and other organs will be evaluated in our future studies.

### Limitations

Our sample sizes were relatively small which could have contributed to some of the negative findings for some of the readouts at some of the time points studied. In addition, we did not analyze correlations between the different parameters that were measured in individual mice as these analyses were not sufficiently powered to detect significant differences. We did not study mice beyond 18 months of age to determine whether the phenotype of the *Cc16* ^−/−^ mice continues to progress after senescence is reached in mice. We also did not confirm that increased spontaneous activation of *NF‐kB* detected in the lungs of *Cc16* ^−/−^ mice is required for their spontaneous development of greater pulmonary inflammation and COPD‐like lung lesions over time. These areas will be the focus of our future studies.

## Conclusions

Our data suggest that CC16 is a circulating factor that protects lungs from aging by restraining lung inflamm‐aging as *Cc16* deficiency accelerates pulmonary inflammation and COPD‐like pulmonary phenotypes in unchallenged mice. Thus, low serum CC16 levels may directly contribute to the impaired lung function that has been detected in human cohorts by increasing pulmonary inflamm‐aging to thereby accelerate the development of senile emphysema and small airway fibrosis which both contribute to airflow obstruction and FEV_1_ decline in humans. If our results are confirmed in human cohorts, our data suggest that CC16 augmentation approaches in young and middle‐aged adults with low serum CC16 levels may have potential as a novel therapeutic approach to preserve lung function in these subjects as they age.

## Conflict of Interest

Dr. Owen is also an employee of Vertex Pharmaceuticals Inc., 50 Northern Avenue, Boston, MA, but has no disclosures relevant to the content of this manuscript.
